# *In Silico* Investigations of the Anti-Catabolic Effects of Pamidronate and Denosumab on Multiple Myeloma-Induced Bone Disease

**DOI:** 10.1371/journal.pone.0044868

**Published:** 2012-09-21

**Authors:** Yan Wang, Bo Lin

**Affiliations:** 1 Department of Infrastructure Engineering, School of Engineering, University of Melbourne, Melbourne, Australia; 2 School of Medicine, Henan University, Kaifeng, China; Memorial Sloan Kettering Cancer Center, United States of America

## Abstract

It is unclear whether the new anti-catabolic agent denosumab represents a viable alternative to the widely used anti-catabolic agent pamidronate in the treatment of Multiple Myeloma (MM)-induced bone disease. This lack of clarity primarily stems from the lack of sufficient clinical investigations, which are costly and time consuming. However, *in silico* investigations require less time and expense, suggesting that they may be a useful complement to traditional clinical investigations. In this paper, we aim to (i) develop integrated computational models that are suitable for investigating the effects of pamidronate and denosumab on MM-induced bone disease and (ii) evaluate the responses to pamidronate and denosumab treatments using these integrated models. To achieve these goals, pharmacokinetic models of pamidronate and denosumab are first developed and then calibrated and validated using different clinical datasets. Next, the integrated computational models are developed by incorporating the simulated transient concentrations of pamidronate and denosumab and simulations of their actions on the MM-bone compartment into the previously proposed MM-bone model. These integrated models are further calibrated and validated by different clinical datasets so that they are suitable to be applied to investigate the responses to the pamidronate and denosumab treatments. Finally, these responses are evaluated by quantifying the bone volume, bone turnover, and MM-cell density. This evaluation identifies four denosumab regimes that potentially produce an overall improved bone-related response compared with the recommended pamidronate regime. This *in silico* investigation supports the idea that denosumab represents an appropriate alternative to pamidronate in the treatment of MM-induced bone disease.

## Introduction

Multiple Myeloma (MM) is a B cell malignancy that is associated with high morbidity and short survival duration after diagnosis. To date, MM remains incurable; therefore, the realistic goal for treating patients with MM is to improve their quality of life and prolong their survival time. Over 70% of patients with MM will develop bone lesions as the MM progress, resulting in osteolytic bone disease that includes severe bone pain, pathological fractures, osteoporosis and hypocalcaemia [1]–[3]. These osteolytic lesions may progress even if patients with MM respond to anti-MM therapy [4], [5]. The bone pain and pathological fractures always cause disability, a loss of independence, and, ultimately, a loss of personal dignity, as well as significantly impact the survival duration [1]. As a result, MM-induced osteolytic bone disease is a major cause of morbidity and mortality in patients with MM [6], and the management of osteolysis in patients with MM is a key aspect in the treatment of this malignancy.

Two categories of agents are used to treat bone disease according to the terminology from [7]: anti-catabolic agents and anabolic agents. The anti-catabolic drugs inhibit osteoclast activity, leading to a slightly increased bone volume with a low bone turnover, whereas the anabolic drugs promote osteoblast activity, resulting in a robust increase in bone volume with a high bone turnover. Whereas anti-catabolic agents are applied in the treatment of MM-induced bone disease, there are currently no anabolic agents that have been approved to treat this condition. In this paper, we focus on investigating the effects of anti-catabolic agents on MM-induced bone disease.

Currently, the most widely used agents to treat MM-induced bone disease are the bisphosphonates, which induce a reduction in both bone resorption and bone turnover through several mechanisms simultaneously (i.e., by inhibiting osteoclast recruitment and accelerating the apoptosis of osteoclasts) [8]. Extensive evidence indicates that pamidronate (a member of the newer generation of bisphosphonates) is effective in the treatment of MM-induced osteolytic bone disease [9]–[11]. Additionally, *in vitro* and *in vivo* experiments support the direct and indirect anti-MM activities of pamidronate, which may include the inhibition of tumor cell functions, the stimulation of anti-tumor immune reactions, and the enhancement of the cytotoxic activity of chemotherapeutic agents [12]–[15]. A regime of consisting of the intravenous administration of 90 mg pamidronate over at least 2 hours every 3 or 4 weeks for a period of 2 years [16] is recommended to treat MM-induced bone disease in the clinical setting. Generally, the patients with MM tolerate pamidronate well; however, renal impairment has been described in patients with MM who had received a prolonged administration of pamidronate [17]. Osteonecrosis of the jaws (ONJ) has been recently reported as a serious but uncommon adverse effect in pamidronate-treated patients, and the incidence of this effect has been reported to increase in an MM group compared with a non-MM group [18].

There is an increasing amount of preclinical and clinical evidence showing that a new, promising anti-catabolic agent, denosumab (AMG 162, a human monoclonal antibody to receptor activator of nuclear factor-κB ligand (RANKL)), is able to improve MM-induced bone disease [19]–[21]. Denosumab has a high affinity and specificity for RANKL [22], with a mean half-life of 33.3 days after the administration of 3 mg/kg denosumab in patients with MM [21]. The most commonly reported adverse events after denosumab administration in patients with MM were anemia, upper respiratory tract infection, fatigue and headache [20]. In addition, a case of ONJ in a patient who had received denosumab was reported very recently [23]. Although denosumab was recently approved to treat osteoporosis [24]–[26] and prevent the skeletal-related events in patients with bone metastases from solid tumors [27], [28] in the United States and Europe, it is still undergoing phase III clinical trials of its efficacy in treating MM-induced bone disease. Without sufficient clinical investigations, it is pre-mature to conclude that denosumab represents a viable alternative to pamidronate in the treatment of MM-induced bone disease.

Although clinical investigations represent a direct and reliable way to determine the efficacy of denosumab, this approach is time-consuming and expensive. Alternatively, *in silico* investigations require less time and money, suggesting that such studies may complement the clinical investigations. We have previously proposed a computational MM-bone model [29] that incorporates the most important mechanisms involved in MM-bone vicious cycles and that has been validated by clinical observations to simulate MM disease progression appropriately. For this reason, the MM-bone model appears to be a suitable computational base for investigating pamidronate and denosumab treatments in MM-induced bone disease. Therefore, the purpose of this paper is to investigate the effects of pamidronate and denosumab on MM-induced bone disease using a computational MM-bone model.

First, we aim to develop integrated computational models suitable for investigating the effects of pamidronate and denosumab on MM-induced bone disease. To achieve this goal, pharmacokinetic models of pamidronate and denosumab are developed to simulate the transient concentrations of pamidronate and denosumab and are calibrated and validated by different clinical datasets. Integrated computational models are then developed by incorporating the simulated transient concentrations of pamidronate and denosumab and simulations of their actions on the MM-bone compartment into the MM-bone model. Additional clinical datasets are used to calibrate and validate the integrated models. With integrated computational models, it is possible to investigate the effects of pamidronate and denosumab on MM-induced bone disease.

The second goal of this paper is to evaluate the *in silico* responses to pamidronate and denosumab treatments, which help clarify whether denosumab represents a useful alternative to pamidronate in the treatment of MM-induced bone disease. The responses to a variety of regimes (including dosages and administration periods) of pamidronate and denosumab treatments in patients with MM are investigated using the validated integrated models. Furthermore, these responses are evaluated by the defined relative response and the relative index, which are based on quantifying the bone volume, bone turnover and MM-cell density. The outcomes of the evaluation may identify certain denosumab regimes that potentially produce an improved bone-related response compared with the recommended pamidronate regime.

This paper is organized as follows. The pharmacokinetic models of pamidronate and denosumab are developed in Section 2.3, the integrated computational models are developed in Section 2.4, and the relative response and the relative index are defined in Section 2.7. In Section 3.1, the pharmacokinetic models of pamidronate and denosumab are calibrated and validated by different clinical datasets, and in Section 3.2, the integrated computational models are calibrated and validated by different clinical datasets. In Section 3.4 and 3.6, the responses to various regimes of pamidronate and denosumab treatments are investigated and evaluated respectively.

## Materials and Methods

### 2.1 Data sources

Table 1 summarizes the clinical datasets used in this paper for the purposes of calibration and validation. One denosumab pharmacokinetic dataset, in which 25 patients with MM received 0.1, 0.3, 1.0, or 3.0 mg/kg denosumab (s.c.) and blood samples of denosumab were collected at 1, 2, 4, 8, and 24 hours after drug administration and at days 1, 2, 3, 4, 8, 15, 22, 29, 43, 57, 71 and 85 [21], was used for the calibration of the denosumab pharmacokinetic model. Another denosumab pharmacokinetic dataset, in which 255 patients with breast-cancer-related bone metastases received 30, 120 or 180 mg denosumab (s.c.) every 4 weeks and blood samples of denosumab were collected at days 8, 29, 57, 71 and 85 [30], was used for the validation of the denosumab pharmacokinetic model. The skeletal retention of pamidronate at days 2, 6, 17 and 28 in 40 patients with breast-cancer-related bone metastasis after receiving 90 mg pamidronate (i.v.) [31] was used for the calibration of the pamidronate pharmacokinetic model. The skeletal retention of pamidronate at days 1, 2, 3, 4 and 5 in 22 patients with osteoporosis after receiving 15 mg pamidronate (i.v.) every day [32] was used for the validation of the pamidronate pharmacokinetic model. Because pharmacokinetics are mainly determined by agents in their own right rather than by the pathological state of patients, it is reasonable to assume that the state of MM, breast-cancer or osteoporosis has no significant impact on the pharmacokinetics of pamidronate and denosumab. Accordingly, the pharmacokinetic datasets of pamidronate and denosumab obtained in patients with bone destruction (i.e., breast-cancer and osteoporosis) but without MM would not significantly influence the reliability of the calibration and validation of the proposed pharmacokinetic models.

**Table 1:** The clinical datasets used for the calibration and validation of the integrated models with pamidronate and denosumab.

		denosumab		pamidronate	
		PK	PD	PK	PD
calibration	input	0.1, 0.3, 1, 3 mg/kg denosumab s.c. in MM for 12 weeks	0.1, 0.3, 1, 3 mg/kg denosumab s.c. in MM for 12 weeks	90 mg pamidronate i.v. in breast cancer for 4 weeks	90 mg pamidronate i.v. in MM for 12 weeks
	output	Denosumab (ng/ml) in serum [21]	NTX (nM) in serum [21]	Pamidronate (mg) in bone [31]	NTX (nM) in serum [21]
	input	NA	NA	NA	Chemotherapy alone or chemotherapy plus 90 mg pamidronate i.v. in MM every month for 14 months
	output	NA	NA	NA	Paraprotein (%) in serum [33]
validation	input	30, 120, 180 mg denosumab s.c. in breast cancer every 4 weeks for 3 months	120 mg denosumab s.c. in relapsed MM every 28 days for 7 months	15 mg pamidronate i.v. in osteoporosis every day for 5 days	90 mg pamidronate i.v. in MM every 4 weeks for 13 months
	output	Denosumab (ng/ml) in serum [30]	CTX (%) in serum [20]	Pamidronate (mg) in bone [32]	NTX (%) in urine [9]

Absolute median serum type I collagen cross-linked N-telopeptides (NTX), collected at days 1, 2, 3, 4, 8, 15, 22, 29, 43, 57, 71 and 85 in 25 patients with MM after receiving 0.1, 0.3, 1.0, or 3.0 mg/kg denosumab (s.c.) or 90 mg pamidronate (i.v.) [21], were used for the calibration of the anti-catabolic effects of the integrated models with denosumab or pamidronate respectively. In particular, the percentage changes of paraprotein from the baseline at months 3, 6, 9, 12 and 14 in 32 patients with MM who received 90 mg pamidronate (i.v.) monthly in combination with chemotherapy or received chemotherapy alone were used for the calibration of the anti-MM effects of the integrated model with pamidronate [33]. The median percentage changes of serum type I collagen cross-linked C-telopeptides (CTX) from the baseline at months 4 and 7 in 53 patients with relapsed MM after receiving 120 mg denosumab (s.c.) at days 1, 8, 15, and 29 and then at day 1 of every cycle (28 days) [20] were used for the validation of the anti-catabolic effects of the integrated model with denosumab. The median percentage changes of urine NTX from the baseline at months 1, 3, 6, 9 and 13 in 167 patients with stage III MM after receiving 90 mg pamidronate (i.v.) every 4 weeks [9] were used for the validation of the anti-catabolic effects of the integrated model with pamidronate. because CTX concentrations are highly correlated with NTX concentrations [34] and measurements of the NTX in the serum reflect bone resorption to the same extent as urinary indices [35], the percentage changes of the CTX and the urinary NTX are assumed to be almost the same as the serum NTX percentage changes. As a result, using CTX and urinary NTX datasets to validate the integrated models with pamidronate and denosumab does not significantly influence the reliability of the validation.

All of the above-described datasets were digitalized from graphs of previously published papers using WinDIG version 2.5.

### 2.2 The structure of the integrated models with pamidronate and denosumab

**Figure 1:**
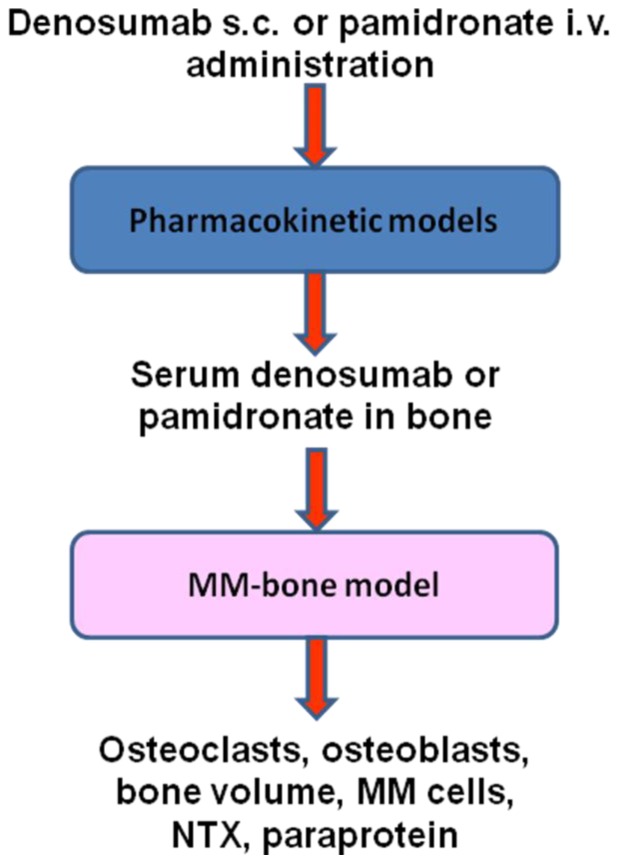
The schematic of the incorporations of the drugs into the MM-bone model. NTX: type I collagen cross-linked N-telopeptides.

We previously proposed a computational MM-bone model [29] that implements the most important mechanisms involved in MM-bone interactions. The dynamic outcomes of this computational model were shown to agree with known clinical observations, suggesting that the two positive feedback cycles identified in this model are sufficient to appropriately replicate MM disease progression. As a result, the MM-bone model appears to be a suitable computational base for investigating the effects of pamidronate and denosumab on MM-induced bone disease. To use the computational MM-bone model to investigate the effects of pamidronate and denosumab on MM-induced bone disease, the agents pamidronate and denosumab must be appropriately integrated into the model. As Figure 1 shows, this integration begins with the pharmacokinetic models of pamidronate and denosumab so that the transient concentrations of pamidronate and denosumab are simulated as inputs into the MM-bone model. After the drugs enter the MM-bone model, the actions of pamidronate and denosumab on the components of the MM-bone model are modeled and incorporated into the MM-bone model. Outputs from the MM-bone model as a result of the pamidronate and denosumab treatments, such as bone resorption marker NTX, are quantified for the purpose of comparing the model with the clinical data. Eventually, integrated computational models incorporating pamidronate and denosumab are produced.

**Figure 2:**
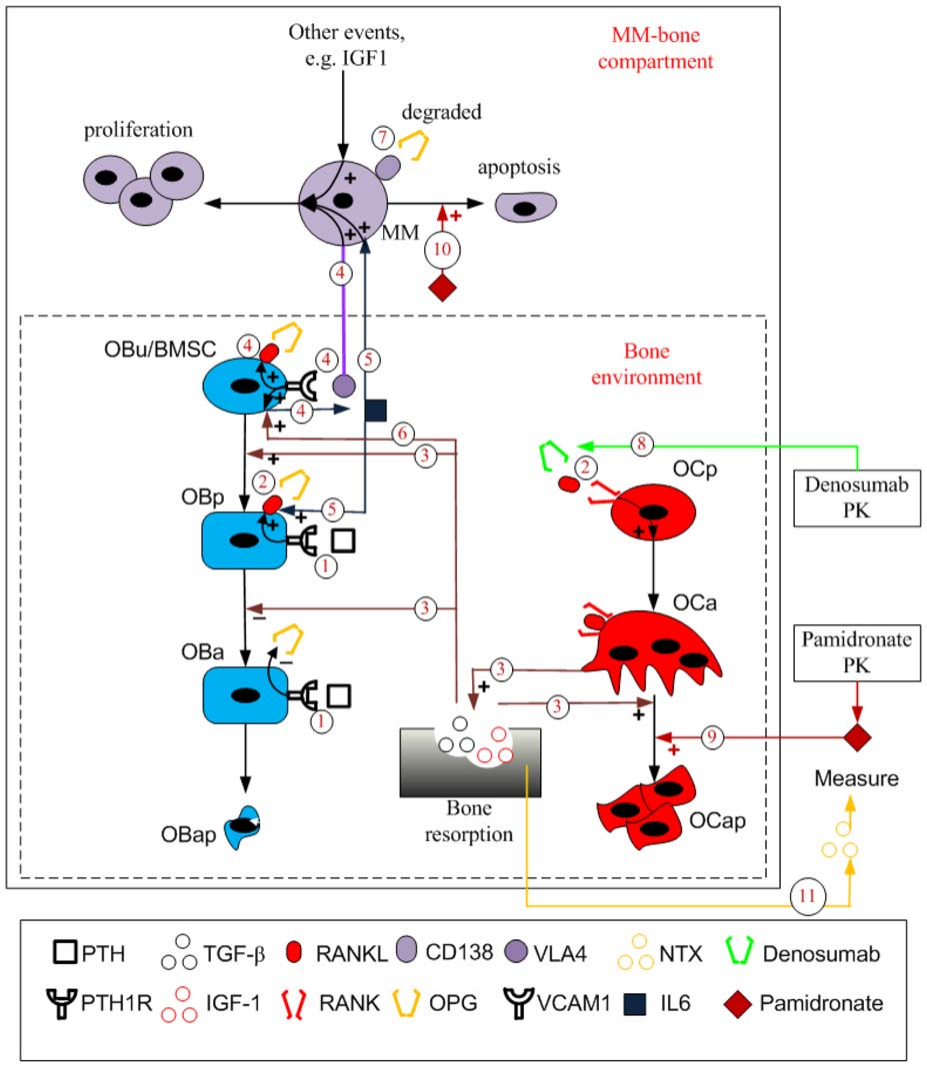
The structure of the integrated models with pamidronate and denosumab. Regulation mechanism 1: PTH stimulates RANKL expression on the surface of osteoblast precursors while inhibiting OPG secretion by active osteoblasts. Regulation mechanism 2: RANKL binds to RANK, which promotes differentiation of osteoclast precursors, while OPG inhibits the RANKL-RANK binding. Regulation mechanism 3: Bone resorption released TGF-β stimulates uncommitted osteoblast differentiation, inhibits osteoblast precursor differentiation and facilitates apoptosis of active osteoclasts. Regulation mechanism 4: MM cells adhere to BMSC, enabling IL-6 secretion by BMSC, RANKL expression on the surface of BMSC and MM-cell proliferation. Regulation mechanism 5: IL-6 facilitates MM-cell proliferation and stimulates RANKL expression on the surface of osteoblast precursors. Regulation mechanism 6: bone resorption released TGF-β stimulates IL-6 production by BMSC. Regulation mechanism 7: OPG is internalized and degraded by MM cells. Regulation mechanism 8: denosumab binds to RANKL to inhibit differentiation of osteoclast precursors. Regulation mechanism 9: pamidronate promotes the apoptosis of active osteoclasts. Regulation mechanism 10: pamidronate facilitates the apoptosis of MM cells. Regulation mechanism 11: NTX released from bone resorption is under the control of active osteoclasts.

Figure 2 illustrates the structure of the integrated computational models. Because the details with respect to the MM-bone model are provided in [29], only the newly introduced drug-related mechanisms in the integrated models are described here. To input the transient concentrations of pamidronate and denosumab into the MM-bone model, two pharmacokinetic models of pamidronate and denosumab are first developed to simulate the transient concentrations of pamidronate and denosumab. By assuming that MM disease has no significant impact on the pharmacokinetics of pamidronate and denosumab, the pharmacokinetic models can be separately simulated within the MM-bone model. As a result, the pharmacokinetic models are run to simulate the transient concentrations of pamidronate and denosumab. During simulations of the MM-bone model, the simulated transient concentrations of pamidronate and denosumab are interpolated and incorporated into the MM-bone model.

After the drugs enter the MM-bone model, the physiological actions of pamidronate and denosumab on the components of the MM-bone model are modeled based on biological evidence, and the modeling of their actions is incorporated into the MM-bone model. As discussed in the Introduction, denosumab is a specific and affinitive human monoclonal antibody to RANKL; therefore, its action on the MM-bone model is modeled as binding to RANKL. This mechanism is incorporated into the MM-bone model as regulation mechanism 8 in Figure 2. A decrease in the density of the osteoclasts caused by pamidronate results from the accelerated apoptosis of the osteoclasts; therefore, this action of pamidronate on the bone is modeled as promoting osteoclast apoptosis, and this mechanism is incorporated into the MM-bone model as regulation mechanism 9 in Figure 2. Additionally, pamidronate exhibits an indirect ability to reduce MM-cell density through various mechanisms. This action of pamidronate on the MM cells is modeled as promoting MM-cell apoptosis, and this mechanism is incorporated into the MM-bone model as regulation mechanism 10 in Figure 2.

Bone-cell densities are a direct way to monitor the imbalance of bone remodeling and to assess the effects of pamidronate and denosumab on MM-induced bone disease; however, only limited and fragmentary clinical data are available in the literature to identify these values [36]. Accordingly, it is impractical to use them to calibrate and validate the integrated models. Alternatively, bone turnover markers (including bone resorption markers and bone formation markers) are usually measured in serum or urine in the clinical setting to indirectly reflect the imbalance of bone remodeling and to assess the effects of drugs on MM-induced bone disease. NTX is one of most commonly used markers to reflect bone resorption activity. To compare the simulation results with the clinical data on NTX, the NTX levels (in serum) need to be linked with bone resorption activity in the computational model. It has been shown that osteoclasts secrete proteases (i.e., Cathepsin) to cleave type I collagen to produce fragments in bone, such as NTX/CTX, that are released into the circulation and cleared by the liver or kidneys [37]. Therefore, as regulation mechanism 11 in Figure 2 shows, the production of NTX is modeled as being under the control of the density of the osteoclasts.

Technically, in this manuscript, the drug treatments are applied in a case of quasi-stable MM (i.e., the proliferation of MM cells *P_MM_* = 0.055/day, see [29]). Given that the median survival duration of the patients with MM after diagnosis is currently in the range 50–55 months [38], the administration of pamidronate and denosumab starts at the end of year-3 of MM disease progression; thus, the state of MM disease at the end of year-3 represents the initial state of the integrated models. It should be noted that another circumstance of administrating drugs at the end of year-4 of MM disease progression has also been examined and it produces consistent results with the circumstance of administrating drugs at the end of year-3 of MM disease progression, suggesting that the results presented in this manuscript are probably irrelevant to the time point of drug administration.

### 2.3 Pharmacokinetic models of pamidronate and denosumab

**Figure 3:**
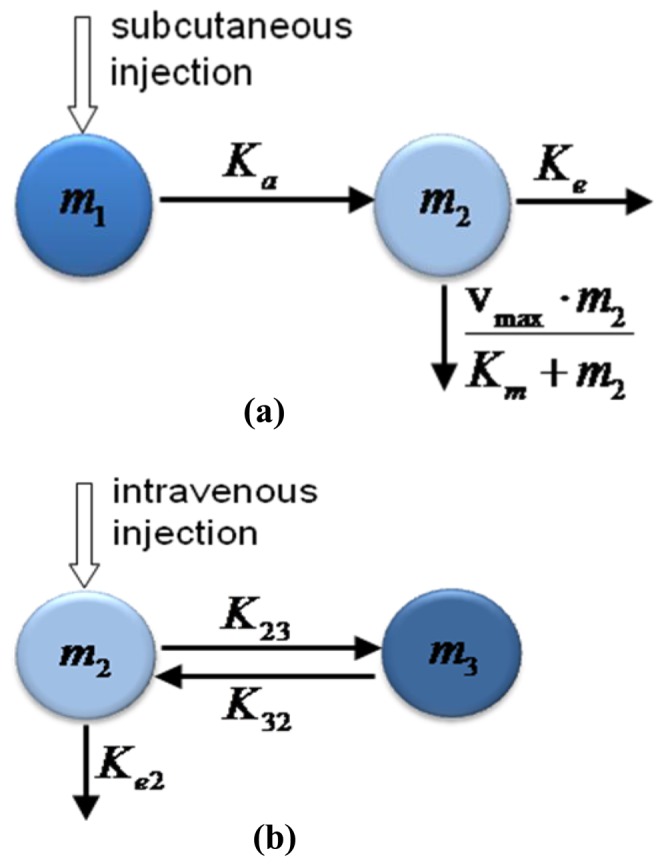
The schematic of the pharmacokinetic models of pamidronate and denosumab. (a) Denosumab. (b) Pamidronate. m_1_: muscle; m_2_: blood; m_3_: bone.

Based on the study of Lipton et al. [30] study, the proposed pharmacokinetic model of denosumab is a two-compartment model with dual first–order and Michaelis–Menten clearances from the blood compartment. As shown in Figure 3a, *m* *_1_* and *m_2_* represent the denosumab concentrations (nM) in muscle tissue after a subcutaneous injection and in blood (nM) respectively; *K_a_* is the absorption of denosumab from the muscle into the blood circulation; *K_e_* denotes the linear elimination constant of denosumab; *K_m_* is the half-maximal concentration of denosumab in Michaelis–Menten elimination; and *V_max_* is the maximal velocity of Michaelis–Menten elimination. The conceptual model describing the pharmacokinetics of denosumab can be expressed as follows:
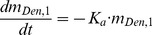
(1)

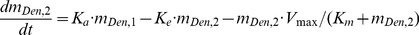
(2)By assuming that pamidronate molecules, both on the surface of the bone and buried within the bone matrix, are acting on osteoclasts, the previously proposed three-compartment model of pamidronate pharmacokinetics [32] is simplified into a two-compartment model. In Figure 3b, *m* *_2_* and *m_3_* represent the pamidronate mass (mg) measured in the blood after an intravenous injection and the retention in the bone (mg) respectively; *K_e2_* is the elimination constant of pamidronate by renal function; and *K_23_* and *K_32_* denote the diffusion constants between *m_2_* and *m_3_* respectively. The corresponding governing equations to describe pamidronate pharmacokinetics are as follows:
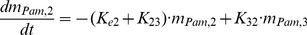
(3)

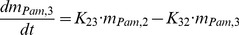
(4)


### 2.4 The formulation of the governing equations of denosumab and pamidronate actions on the MM-bone model

Without drug treatments, the concentrations of RANKL are calculated as follows [29]:
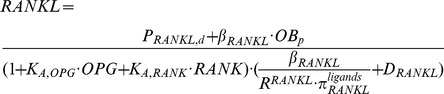
(5)The binding of denosumab to RANKL influences the concentrations of RANKL; therefore, with denosumab treatments, equation (5) to calculate the concentrations of RANKL is replaced by the following:
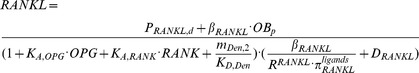
(6)where, *m_Den,2_* is the denosumab concentration in serum and *K_D,Den_* is proportional to the dissociation rate constant of denosumab binding to RANKL (the derivation of *K_D,Den_* is described in the Supporting Information S1). *K_A,OPG_* and *K_A,RANK_* are the association rate constant of RANKL binding to osteoprotegerin (OPG) and receptor activator of nuclear factor-κB (RANK) respectively. *R^RANKL^* is the maximal number of RANKL molecules that can be expressed on the surface of osteoblast precursors. *P_RANKL,d_* is the external production rate of RANKL with the unit of 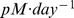. *β_RANKL_* is the endogenous production of RANKL by osteoblast precursors with the unit of 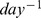. *D_RANKL_* is the degradation of RANKL. 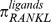 is the enhanced ‘activator’ function in response to simultaneous parathyroid hormone (PTH) and IL-6 stimulations.

Without drug treatments, the population of active osteoclasts and MM cells are calculated respectively as follows [29]:
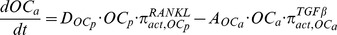
(7)

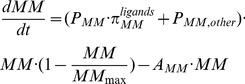
(8)With pamidronate treatments, the apoptosis of active osteoclasts is co-regulated by simultaneous transforming growth factor β (TGF-β) and pamidronate stimulations. Using equation (3) in [29], which models the ‘enhanced’ cellular response to simultaneous two ligand stimulations, the suggested action of pamidronate on the bone microenvironment is modeled. As such, the governing equation of the population of active osteoclasts (equation (7)) is replaced by the following equations:
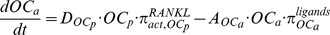
(9)

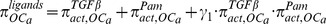
(10)

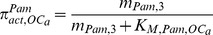
(11)where, *m_Pam,3_* is the pamidronate concentration retained in the bone. *K_M,Pam,OCa_* is the half-maximal concentration of pamidronate to kill active osteoclasts. 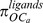 is an ‘activator’ function, which models the apoptosis of active osteoclasts in response to simultaneous TGF-β and pamidronate stimulations. 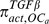 and 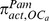 are the ‘activator’ functions of the apoptosis of active osteoclasts under the control of TGF-β and pamidronate respectively. *γ_1_* is a parameter that can be calibrated to reflect the effects of the co-stimulation with TGF-β and pamidronate.

In addition, using equation (3) in [29], we incorporate the suggested action of pamidronate on MM cells into equation (8) as follows:
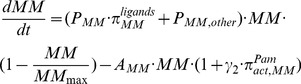
(12)

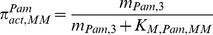
(13)where, *m_Pam,3_* is the pamidronate concentration retained in the bone; *K_M,Pam,MM_* is the half-maximal concentration of pamidronate to kill MM cells; 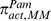 is the ‘activator’ function of pamidronate promoting the apoptosis of MM cells; and *γ_2_* is a parameter that can be calibrated to reflect the effects of pamidronate stimulation. In this analysis, *γ_2_* is set to 1 to simplify the integrated model.

Finally, the bone volume as a system output of the integrated models is calculated as follows [29]:
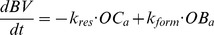
(14)where *BV* represents normalized bone volume, *k_res_* and *k_form_* represent relative rate of bone resorption and bone formation respectively with the unit of 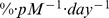.

### 2.5 The formulation of the governing equations of NTX output

Because the production of NTX from type I collagen is controlled by the density of the active osteoclasts and serum NTX is cleared by the liver or kidneys, the changes of the serum NTX over time compose one source term (i.e., the production of NTX in the bone) and one sink term (i.e., the degradation of NTX in the bone and blood). Here, we assume that the concentrations of type I collagen are a constant because they are much greater than the NTX concentrations in the bone. In addition, we assume the following: (i) the production of NTX is controlled by the density of active osteoclasts in the form of the Hill function, and (ii) NTX degrades at the first–order kinetics in the serum. The changes of the serum NTX over time are summarized as follows:
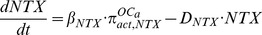
(15)

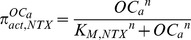
(16)where, *β_NTX_* denotes the production rate of NTX and *D_NTX_* denotes the degradation of NTX. 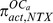 denotes the ‘activator’ function of active osteoclasts stimulating the production of NTX. *K_M,NTX_* is the half-maximal density of active osteoclasts to control the production of NTX, and *n* is the Hill coefficient.

### 2.6 Calibration of the parameters

The calibration of the parameters is performed using the least-square criterion, that is, to minimize the following objective function:
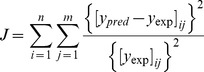
(17)where, *y_pred_* and *y_exp_* are simulated values and the corresponding experimental or clinical values respectively. The values of *y_pred_* are calculated using the routine ‘ode15s’ in Matlab, and the values of *y_exp_* are collected from the literature (see Table 1). Additionally, n is the number of experiments, and m is the number of sampled points in each experiment. The optimization process is implemented using the routine ‘patternsearch’ in Matlab.

To evaluate the goodness of fit between simulations and experiments, the coefficient of determination (r^2^) is defined as follows:
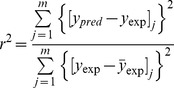
(18)where, 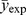 is the mean of *y_exp_*. The closer this value is to 1, the better the agreement is between the simulation results and the experimental data.

### 2.7 The quantification of the responses to pamidronate and denosumab treatments in patients with multiple myeloma

In a clinical situation, the primary concerns regarding pamidronate and denosumab treatments in MM-induced bone disease are the extent to which these treatments improve osteolytic bone lesions and whether these treatments cause serious adverse effects. Additionally, the extent to which these treatments alleviate the MM tumor burden is of concern, although pamidronate and denosumab are basically considered anti-catabolic agents. One of reasons for this concern is that anti-catabolic agents are found to inhibit MM-cell proliferation and survival [39], [40]. To link the *in silico* investigations of pamidronate and denosumab treatments in MM-induced bone disease with clinical concerns, *in silico* investigations should provide all of the above-mentioned information.

Changes over time in the active osteoblast and osteoclast populations, relative to each other, result in changes in the bone volume, which is a ‘system output’ of the bone compartment [29]. Consequently, bone volume appears to be an appropriate variable for tracking the improvements in osteolytic bone lesions. As a state variable of the proposed integrated models, MM-cell density appears to be an appropriate variable for tracking the alleviation of the MM tumor burden. Unfortunately, it is impossible for *in silico* investigations to track various adverse effects because these effects are associated with a number of other organs, which is beyond the scope of the MM-bone model. However, it is possible for *in silico* investigations to track specific adverse effects. For example, ONJ is found to be a serious adverse effect of pamidronate treatments. Although the exact mechanisms of ONJ occurrence are unknown, the inhibition of osteoclast function and differentiation might be a key factor [23]. Accordingly, the density of osteoclasts appears to be an appropriate variable for tracking the occurrence of ONJ, which is an output from the proposed integrated models.

Furthermore, after pamidronate and denosumab treatments the accumulated absolute deviation of these variables (i.e., bone volume, MM-cell density and the density of osteoclasts) over time can be quantified by calculating the area under their curves (AUC), which is the time integral of the change in the variable from the beginning of the treatment to the end of the treatment. Supporting Information S2 illustrates the specific calculations of the AUCs for the bone volume, the MM-cell density and the density of osteoclasts (denoted by *BV_AUC_*, *MM_AUC_* and *OCa,_AUC_* respectively). Higher values of *BV_AUC_*, *MM_AUC_* and *OCa,_AUC_* indicate a greater improvement in MM-induced bone lesions, a greater alleviation of the MM tumor burden and a reduced occurrence of ONJ respectively.

Pamidronate has been used in clinical settings to treat MM-induced bone disease for many years, and a regime consisting of the intravenous administration of 90 mg pamidronate every 4 weeks for a period of 2 years is recommended. In contrast, denosumab is a novel agent that is still undergoing the phase III clinical trials to treat MM-induced bone disease; accordingly, different denosumab regimes are undergoing testing, and no recommended denosumab regimes are available yet to treat MM-induced bone disease. In the meanwhile, according to the highlights of prescribing information provided by Amgen Inc. (http://pi.amgen.com/united_states/xgeva/xgeva_pi.pdf), it is worth mentioning that the administration of 120 mg denosumab every 4 weeks has been recommended to treat the skeletal-related events in patients with bone metastases from solid tumors although there is no experience of over-dosage of denosumab. For this reason, the above mentioned denosumab regime can be probably regarded as a reference regime rather than a recommended regime to treat MM-induced bone disease before more clinical data are available. To evaluate the responses to denosumab treatments in MM-induced bone disease, the pamidronate treatment with the recommended regime is selected as a base treatment. By doing so, the relative response to the denosumab treatment compared with the response to the pamidronate treatment is calculated (denoted by *BV_AUC,rela_*, *MM_AUC,rela_* and *OCa,_AUC,rela_* respectively); that is, the ratio of the responses to various tested denosumab regimes to the responses to the recommended pamidronate regime is calculated. If the relative response is greater than 1, then the denosumab regime appears to produce a better response than the recommended pamidronate regime; otherwise, the denosumab regime is interpreted to produce a response inferior to that of the recommended pamidronate regime.

While *MM_AUC,rela_* is a single criterion to evaluate the MM tumor-related response, *BV_AUC,rela_* and *OCa,_AUC,rela_* are two separate criteria to evaluate the bone-related response. To use a single criterion to evaluate the bone-related response to pamidronate and denosumab treatments rather than using two criteria respectively, a relative index (*Index_rela_*) is defined in equation (19) to evaluate the bone-related response to the pamidronate and denosumab treatments by combining the relative responses of the bone volume and the density of osteoclasts.
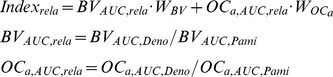
(19)Here, *BV_AUC,rela_* and *OCa,_AUC,rela_* represent the responses of the bone volume and the density of osteoclasts to denosumab treatments relative to their responses to pamidronate treatments respectively. The definitions and calculations of *BV_AUC,Deno_*, *OCa,_AUC,Deno_*, *BV_AUC,Pami_* and *OCa,_AUC,Pami_* are described in Supporting Information S2. *W_BV_* and *W_OCa_* represent the weights of the bone volume and the density of osteoclasts to the bone-related response. The density of osteoclasts is assumed to be a less important criterion compared with the bone volume because it is associated with the occurrence of ONJ that is uncommon after pamidronate and denosumab treatments. Specifically, the risk of ONJ associated with pamidronate is 1 to 2% within the first 2 years of the treatment [41]. Therefore, the weights *W_BV_*, and *W_OCa_* are set to 1and 0.1 respectively according to their relative importance. It should be noted that different evaluation outcomes may be produced when different weights are used. Given that there is no more knowledge to help determine these weights more accurately so far, it is particularly important to experimentally validate the evaluation outcomes that are produced under the current setting of weights.

A greater value of the relative index indicates a better bone-related response to the denosumab regimes compared with the recommended pamidronate regime. Clearly, the thresholds of the *Index_rela_* is1.1; that is, if the value of the *Index_rela_* is greater than 1.1, the denosumab regimes would produce a better bone-related response than the recommended pamidronate regime.

## Results

### 3.1 The calibration and validation of the pharmacokinetic models of pamidronate and denosumab

**Figure 4:**
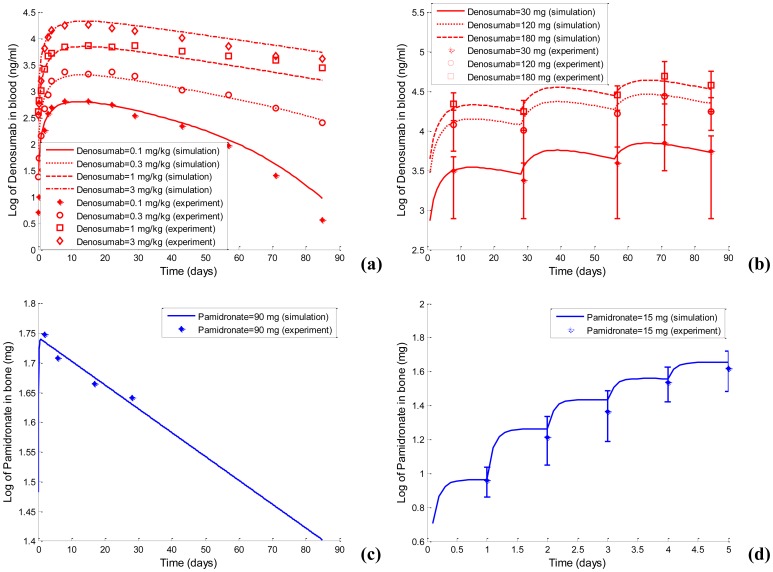
The calibration and validation of the pharmacokinetic models of pamidronate and denosumab. (a) The calibration of the pharmacokinetic model of denosumab. (b) The validation of the pharmacokinetic model of denosumab. (c) The calibration of the pharmacokinetic model of pamidronate. (d) The validation of the pharmacokinetic model of pamidronate.

Based on the clinical pharmacokinetic datasets of denosumab in the blood and pamidronate in the bone after a single-dose administration, the optimized pharmacokinetic models of pamidronate and denosumab are obtained, and the optimized parameter values are listed in Table 2. As displayed in Figure 4, the simulated concentrations of denosumab and pamidronate using the optimized parameter values fit very well with the clinical datasets. The predicted concentrations of denosumab and pamidronate after a multi-dose administration also fit very well with additional clinical datasets. Consistently, as the r^2^ values listed in Table 3 indicate, the simulated concentrations of denosumab and pamidronate closely agree with the concentrations measured in clinical settings under both single-dose and multi-dose administrations. As a result, the optimized pharmacokinetic models of pamidronate and denosumab are suitable for simulating the transient concentrations of pamidronate and denosumab, which are incorporated into the MM-bone model to examine the responses to pamidronate and denosumab treatments in MM-induced bone disease.

**Table 2:** The calibrated parameter values of the pharmacokinetic models of pamidronate and denosumab.

Parameter	Value	Unit	Description
K_a_	1.73e-1	/day	The absorption of denosumab from the muscle
K_e_	2.02e-2	/day	The linear elimination of denosumab
K_m_	5e-1	nM	Half-maximal concentrations of denosumab in Michaelis–Menten elimination
V_max_	5.6e-2	nM/day	The maximal velocity of Michaelis–Menten elimination
V_c_F	1.06e-1	l/kg	The factor that transforms the dosage from mg/kg into mg/l
K_e2_	3.05	/day	The elimination of pamidronate by the kidneys
K_23_	4.89	/day	The diffusion of pamidronate from the blood into the bone
K_32_	2.41e-2	/day	The diffusion of pamidronate from the bone into the blood

**Table 3:** Evaluations of the calibration and validation of pharmacokinetic models of pamidronate and denosumab.

	Calibration				Validation		
Drug	Denosumab (mg/kg)				Denosumab (mg) administered every 4 weeks		
Dose	0.1	0.3	1	3	30	120	180
r^2^	0.9535	0.9157	0.8621	0.8598	0.9061	0.8432	0.9249

Drug	Pamidronate (mg)	Pamidronate (mg) administered every day
Dose	90	15
r^2^	0.9968	0.9701

### 3.2 The calibration and validation of the integrated models with pamidronate and denosumab

The parameters related to the anti-catabolic action of pamidronate and denosumab (*K_D,Deno_*, *K_M,Pami,OCa_* and *γ_1_*) are calibrated by best fitting with the serum NTX concentrations measured in the clinical setting. By assuming that the serum paraprotein changes are proportional to the changes in MM-cell density, the parameter related to the anti-MM action of pamidronate (*K_M,Pami,MM_*) is calibrated by best fitting with the serum paraprotein concentrations measured in the clinical setting. The original dataset of the paraprotein concentrations (represented as percentage changes from the baseline) are obtained in patients with MM who received pamidronate in combination with chemotherapy or received chemotherapy alone [33]. The calibration of the parameter *K_M,Pami,MM_* requires the dataset obtained in patients with MM who received pamidronate alone. To account for this difference, we assume that the effects of pamidronate and chemotherapy on the changes in paraprotein concentrations are linear addition. Therefore, the difference between paraprotein changes after the simultaneous pamidronate and chemotherapy treatment (denoted by paraproteinchanges_chemo+pami_) and those after the chemotherapy treatment alone (denoted by paraproteinchanges_chemo_) is the paraprotein changes after the pamidronate treatment alone (denoted by paraproteinchanges_pami_); that is, paraproteinchanges_pami_ = paraproteinchanges_chemo+pami_ – paraproteinchanges_chemo_. Given that the two curves (paraproteinchanges_chemo+pami_ and paraproteinchanges_chemo_) have some scatter, they are firstly smoothed and then used to calculate the difference between them. The details of smoothing the two curves are described in the Supporting Information S3. We are aware that the assumption and derivation potentially induce an inaccuracy in the calibration. However, the consequences of an inaccurate calibration are mainly limited to the evaluation on the changes in MM-cell density because the anti-MM action of pamidronate appears to have no significant impact on the bone volume (see Section 3.3).

**Figure 5:**
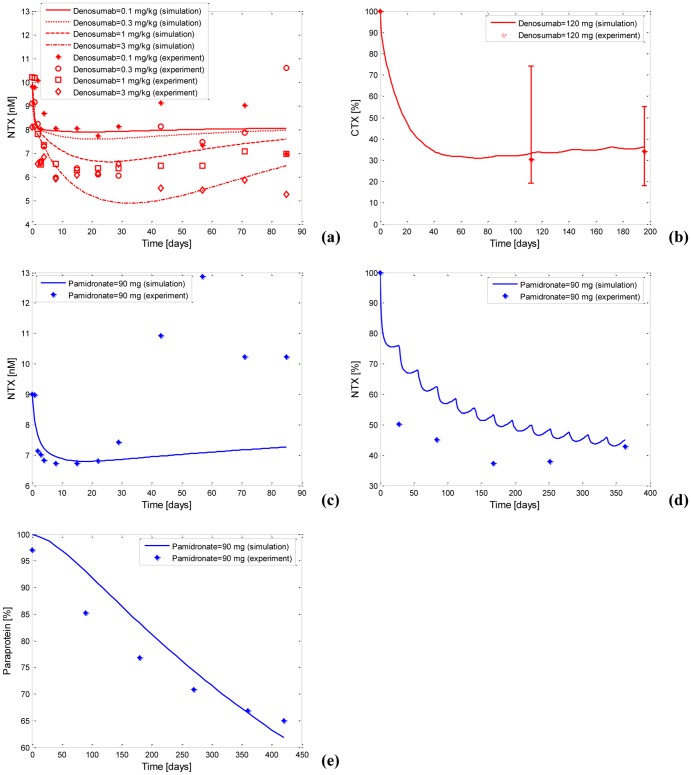
The calibration and validation of the integrated computational models with pamidronate and denosumab. (a) The calibration of the integrated model with denosumab according to the NTX levels. (b) The validation of the integrated model with denosumab according to the CTX levels. (c) The calibration of the integrated model with pamidronate according to the NTX levels. (d) The validation of the integrated model with pamidronate according to the NTX levels. (e) The calibration of the integrated model with pamidronate according to the paraprotein levels.

Table 4 lists the calibrated parameter values of the integrated computational models. Figure 5 displays the fittings between the simulated NTX (and paraprotein) concentrations using the calibrated parameter values and the NTX (and paraprotein) concentrations measured in the clinic. Table 5 summarizes the evaluations of the goodness of fit of these simulations.

**Table 4:** The calibrated parameter values of the integrated models with pamidronate and denosumab.

Parameter	Value	Unit	Description
K_D,Den_	1.45e+4	pM	The dissociation rate constant of denosumab binding to RANKL
K_M,Pam,OCa_	6.04e+2	mg	Half-maximal concentrations of pamidronate to kill active osteoclasts
γ_1_	4	-	A constant to reflect the co-stimulation of TGF-β and pamidronate on the apoptosis of active osteoclasts
K_M,Pam,MM_	9e+1	mg	Half-maximal concentrations of pamidronate to kill MM cells
γ_2_	1	-	A constant to reflect the pamidronate stimulation on the apoptosis of MM cells
β_NTX_	2.31e+4	pM/day	The production rate of NTX
D_NTX_	1.5	/day	The degradation of NTX
K_M,OCa,NTX_	1.8e-4	pM	Half-maximal density of active osteoclasts to control the production of NTX
n	7e-1	-	Hill coefficient

**Table 5:** Evaluations of the calibration and validation of the integrated models with pamidronate and denosumab.

	Calibration				Validation
Drug	Denosumab (mg/kg)				Denosumab (mg) administered every 28 days in relapsed MM
Dose	0.1	0.3	1	3	120
r^2^	0.1137	0.1902	0.6913	0.1181	0.9955

Drug	Pamidronate (mg)	Pamidronate (mg), every 4 weeks administration	Pamidronate (mg) administered every 4 weeks
Dose	90	90	90
r^2^	0.7563^1^	0.0.8199^2^	0.5287

Note 1: The r^2^ is calculated before 30 days after pamidronate treatment.

Note 2: The r^2^ is calculated to evaluate the goodness of fit paraprotein concentrations.

As Figure 5a shows, the simulated NTX concentrations after single-dose denosumab administration exhibit the same trends as the clinical NTX concentrations, but the quantitative evaluations (Table 5) indicate that the simulated NTX concentrations only moderately agree with the clinical data. A possible reason may result from the variation in the clinical pharmacodynamic datasets used in the fittings. In contrast, the agreements between the predicted NTX concentrations and the additional clinical dataset after multi-dose denosumab administration are improved, as indicated in Figure 5b and Table 5. This performance of the calibration and validation suggests that the integrated model with denosumab has a good ability to predict, whereas its ability to fit is moderate.

Furthermore, the simulated NTX concentrations after single-dose pamidronate administration show a close agreement with the clinical NTX concentrations before 30 days. However, the agreement deteriorates after 30 days because the NTX in the simulated curve appears to decrease sustainably, whereas it rebounds after 30 days in the clinical data (Figure 5c). In fact, both the sustained decreases in NTX and the rebounding decreases in NTX after bisphosphonates therapy are observed in the clinic. For example, after single-dose alendronate (a similar agent to pamidronate) administration, NTX levels either sustainably drop in some patients with MM, or decrease and rebound after 7 days of treatment and decrease without any treatment in other patients with MM [42]. Although the sustained decrease in NTX appears to be associated with the long-term effects of the pamidronate embedded in the bone [8], the reason for the rebound behavior of NTX concentrations is not known. The integrated model with pamidronate appears to capture the sustained decrease in NTX concentrations, but does not capture the rebound behavior of NTX concentrations because the mechanisms are unclear and are not incorporated in the integrated model with pamidronate. In spite of the inability to capture the rebound behavior of the NTX concentrations, the predicted NTX concentrations after multi-dose pamidronate administration agree well with the additional clinical dataset, as shown in Figure 5d and Table 5. Additionally, Figure 5e indicates that the simulated percentage changes in the paraprotein after multi-dose pamidronate administration fit well with the clinical data.

Taken together, the calibrated integrated models exhibit a good ability to predict the long-term responses to multi-dose pamidronate and denosumab treatment in patients with MM, although their ability to fit to the short-term responses to single-dose pamidronate and denosumab treatment is only moderate. Consequently, the validated integrated models are suitable to investigate the responses (especially the long-term responses after multi-dose administration) to pamidronate and denosumab treatments in MM-induced bone disease.

### 3.3 Does the anti-MM action of pamidronate have a significant impact on the bone volume?

In the integrated computational models, the actions of pamidronate are modeled as both anti-catabolic and anti-MM, and the action of denosumab is modeled as anti-catabolic. As a result of this difference, the issue of whether the anti-MM action of pamidronate has a significant impact on the bone volume arises. To address this issue, the AUCs of the integrated model with pamidronate are compared with those of the integrated model with pamidronate in which the anti-MM action of pamidronate is disabled (that is, regulation mechanism 10 in Figure 2 is blocked). During the comparisons, the same regimes (the same dosages and administration periods) are used, and the common parameter values used in the intact and blocked integrated models with pamidronate are the same. Paired samples *t*-tests are conducted in SPSS (version 19) to evaluate whether the impact of the anti-MM action of pamidronate on the bone volume is significant, in which the threshold of significance values is set to 0.1.

**Figure 6:**
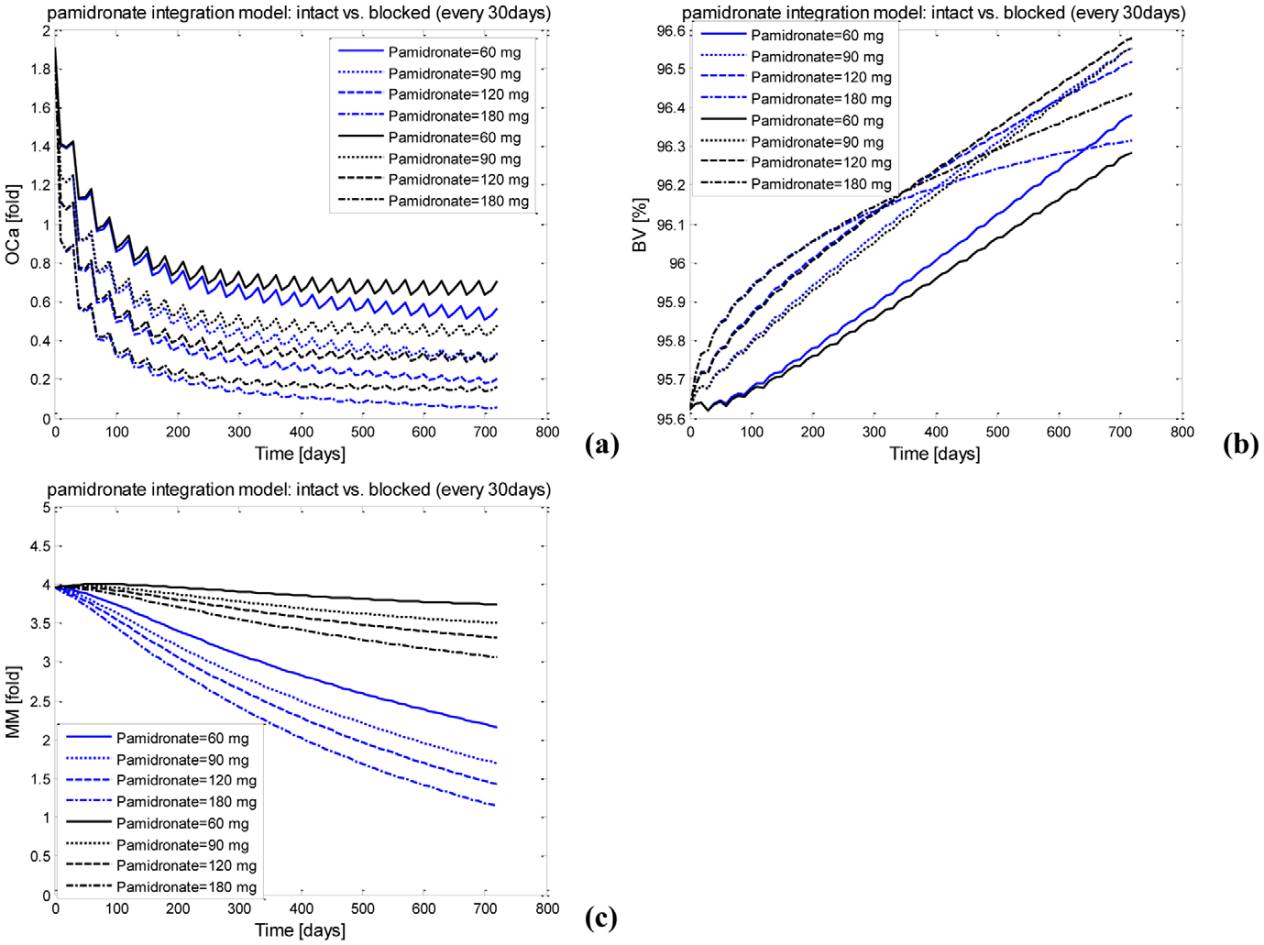
A comparison of the responses to the intact pamidronate integrated model with those to the blocked pamidronate integrated model after multi-dose pamidronate administration. (a) The active osteoclast after multi-dose pamidronate administration. (b) The bone volume after multi-dose pamidronate administration. (c) The MM-cell density after multi-dose pamidronate administration. Blue: the intact model; Black: the blocked model.

After various multi-dose pamidronate administrations, the blocked integrated model with pamidronate induces the AUCs of the density of osteoclasts to increase (Figure 6a) but the AUCs of MM-cell density to decrease (Figure 6c) in comparison to the intact pamidronate integrated model. The AUCs of the bone volume either increase or decrease in the blocked pamidronate integrated model compared with the intact pamidronate integrated model (Figure 6b). While the significance values of the blocked and intact pairs with regard to the density of osteoclasts and MM-cell density are lower than 0.1, the significance value (0.953) of the blocked and intact pair with regard to the bone volume is greater than 0.1. These significance values indicate that the anti-MM action of pamidronate appears to have no significant impact on the bone volume. This clarification helps evaluate the bone-related response to pamidronate and denosumab treatments more accurately.

### 3.4 Investigations of the effects of pamidronate and denosumab on patients with multiple myeloma

**Figure 7:**
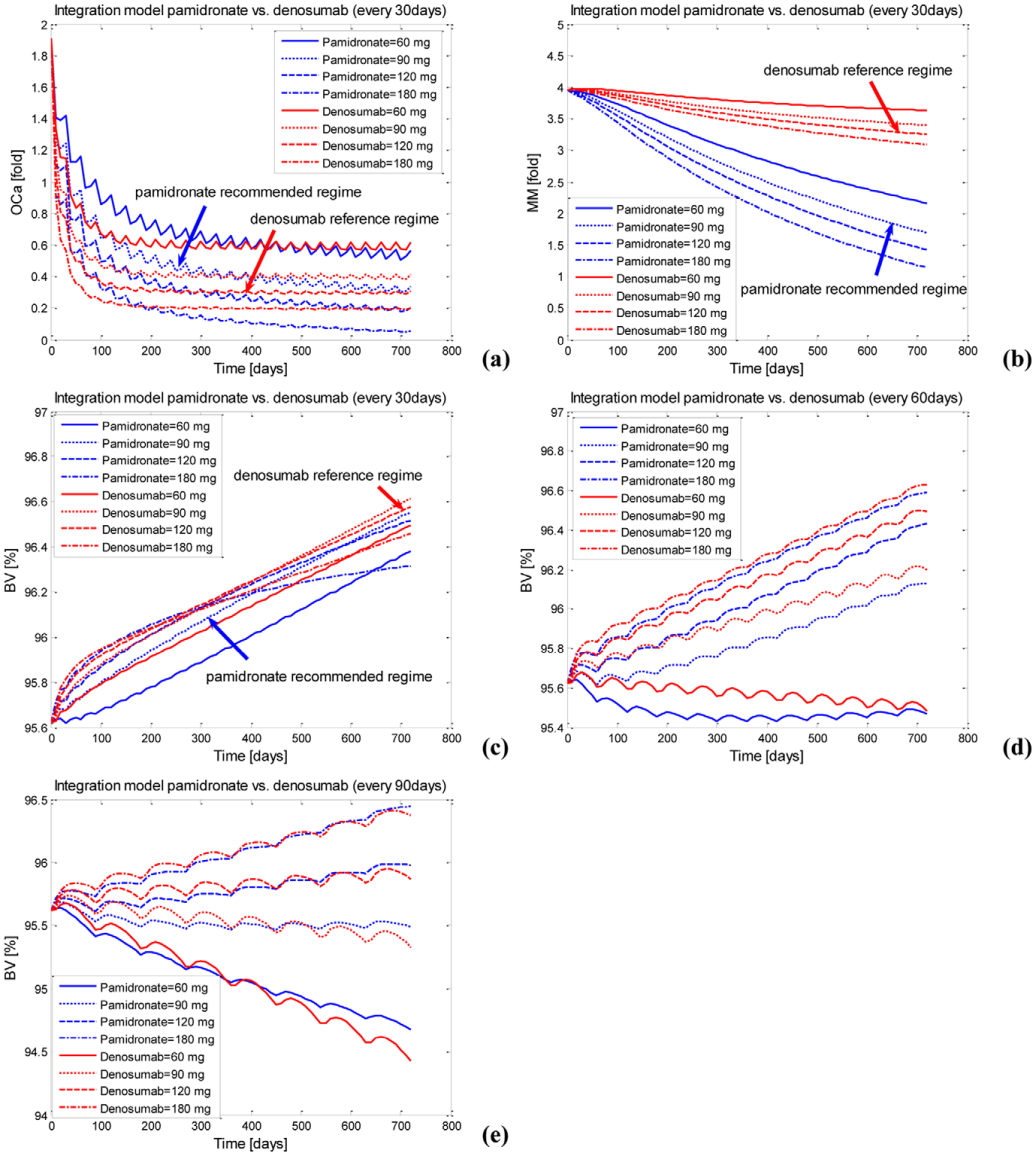
The responses to the integrated models after various regimes of denosumab and pamidronate administration. (a) The active osteoclasts after multi–dose administration every 30 days. (b) The MM-cell density after multi-dose administration every 30 days. (c) The bone volume after multi-dose administration every 30 days. (d) The bone volume after multi-dose administration every 60 days. (e) The bone volume after multi-dose administration every 90 days.

A wide range of regimes of pamidronate and denosumab treatments (dosages of 60, 90, 120 or 180 mg and administration periods of 30, 60 or 90 days, resulting in 12 different regimes) are investigated using the validated integrated models. After various multi-dose administrations of pamidronate and denosumab every 30, 60 or 90 days, there are similar responses of the density of osteoclasts and the MM-cell density. For example, the density of osteoclasts decreases under all regimes of pamidronate and denosumab. The greater dosages of pamidronate and denosumab show greater decreases in the density of osteoclasts (Figure 7a). It should be noted that the curves of the density of osteoclasts exhibit a decrease tendency after pamidronate treatments, which is probably caused by the long-term embedding of pamidronate in the bone. The MM-cell density also decreases under all regimes of pamidronate and denosumab. The greater the dosage of pamidronate and denosumab is, the greater the decrease in the MM-cell density (Figure 7b). It is clear that the great decrease in MM-cell density after pamidronate treatments are caused by the action of pamidronate on killing MM cells while the slight decrease in MM-cell density after denosumab treatments result from the indirect action of denosumab on MM cells through a complicate pathway. As Figure 2 shows, denosumab treatments induce a decreased density of osteoclasts and followed by a decreased TGF-β release from bone resorption, which leads to a decreased IL-6 production by BMSC. Eventually, the decrease in IL-6 concentrations cause a decreased proliferation of MM cells and hence a decreased MM-cell density.

In contrast, the bone volume responds differently to various multi-dose administrations of pamidronate and denosumab every 30, 60 or 90 days. For example, after various multi-dose administrations of pamidronate and denosumab every 30 days, the bone volume increases under all regimes of pamidronate and denosumab. However, the rates of increase in the bone volume drop faster with the increases in the dosage of pamidronate and denosumab (Figure 7c). In particular, the bone volume tends towards plateau at a lower level after 180 mg pamidronate is administrated every 30 days. In this circumstance, the density of active osteoclasts decreases below 0.1-fold (Figure 7a) and the density of active osteoblasts also decreases below 0.1-fold (figure not shown); therefore, this tendency of the bone volume towards plateau probably results from the very low rate of bone turnover. When various multi-dose administrations of pamidronate and denosumab are given every 60 or 90 days, the bone volume either increases or decreases depending on the dosage of pamidronate and denosumab, and greater dosages of pamidronate and denosumab lead to greater gains in the bone volume (Figure 7d–e).

The responses to the recommended pamidronate regime (90 mg administration every 4 weeks) and the reference denosumab regime (120 mg administration every 4 weeks) are highlighted in Figure 7a–c respectively. Compared with the recommended regime of pamidronate, the reference regime of denosumab induces a greater increase in the bone volume, a greater decrease in the density of osteoclasts and a smaller decrease in the density of MM-cells.

### 3.5 What factors mainly contribute to the bone-related response to pamidronate and denosumab treatments?

The above investigations have indicated that pamidronate and denosumab have quite different effects on the MM-cell density and the reason is clear that pamidronate has a direct influence on the MM-cell density while denosumab indirectly influences the MM-cell density. In terms of the bone-related response to pamidronate and denosumab treatments, the two drugs qualitatively produce similar effects because both of them reduce the production rate of active osteoclasts (decrease the right-hand-side of Eq.(9)); although denosumab achieves this by inhibiting the differentiation of osteoclast precursors (decrease the first positive item of right-hand-side of Eq.(9)) while pamidronate achieves this by promoting the apoptosis of active osteoclasts (increase the second negative item of right-hand-side of Eq.(9)). However, the two drugs quantitatively produce different effects; for example, the reference denosumab regime induces a greater increase in the bone volume, a greater decrease in the density of osteoclasts and a smaller decrease in the density of MM-cells than the recommended pamidronate regime. The different quantitative bone-related response is probably mainly attributed to the different pharmacokinetics of pamidronate and denosumab; that is, denosumab has a long half-life in serum while pamidronate has a quite short half-life in serum but embeds in the bone for a long term.

To check whether the allowable dosage of one of pamidronate and denosumab gives a higher bone-related response than the other, we use the MM-bone model (rather than the integrated models) to investigate the responses to pamidronate and denosumab treatments when their different pharmacokinetics are removed. For example, we simulate the responses to denosumab treatments by increasing the value of *D_OCp_* at the beginning of the fourth year of MM progression in the bone because denosumab plays its role by decreasing the first positive item of right-hand-side of Eq.(7). Similarly, we simulate the responses to pamidronate treatments by decreasing the value of *A_OCa_*. The amounts of the increase in *D_OCp_* and the decrease in *A_OCa_* are proportional to the dosages of denosumab and pamidronate respectively. In addition, the upper limit of the increase in *D_OCp_* and the lower limit of the decrease in *A_OCa_*, which induce the strongest responses to denosumab and pamidronate treatments respectively, are determined by the decrease in the density of osteoclasts, which is assumed to at most decrease 50% in these simulations.

**Figure 8:**
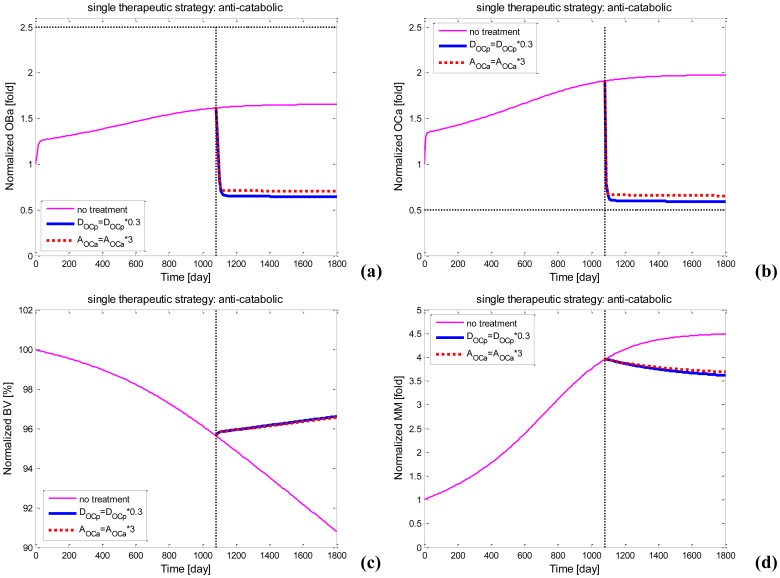
Changes in the density of bone cells, bone volume and the density of MM cells after single anti-catabolic drug treatments. (a) Changes in osteoblast density after therapies; (b) Changes in osteoclast density after therapies; (c) Changes in bone volume after therapies; (d) Changes in MM-cell density after therapies.

One example of almost the strongest responses to denosumab and pamidronate treatments is shown in Figure 8. It clearly indicates that pamidronate and denosumab have the ability to produce almost the same effects on the decrease in the density of osteoblasts and osteoclasts, the decrease in the MM-cells density and the increase in the bone volume. Therefore, the possibility that the allowable dosage of one of pamidronate and denosumab gives a higher bone-related response than the other can be excluded. In addition, pamidronate and denosumab are not involved in other items of the MM-bone model except for the involvement in the right-hand-side of Eq.(9); therefore, the possibility that other items of the MM-bone model cause the different quantitative bone-related response to pamidronate and denosumab treatments can also be excluded. In a summary, the different pharmacokinetics of pamidronate and denosumab mainly contribute to the different quantitative bone-related response to pamidronate and denosumab treatments.

### 3.6 Is denosumab an alternative to pamidronate in the treatment of MM-induced bone disease?

**Figure 9:**
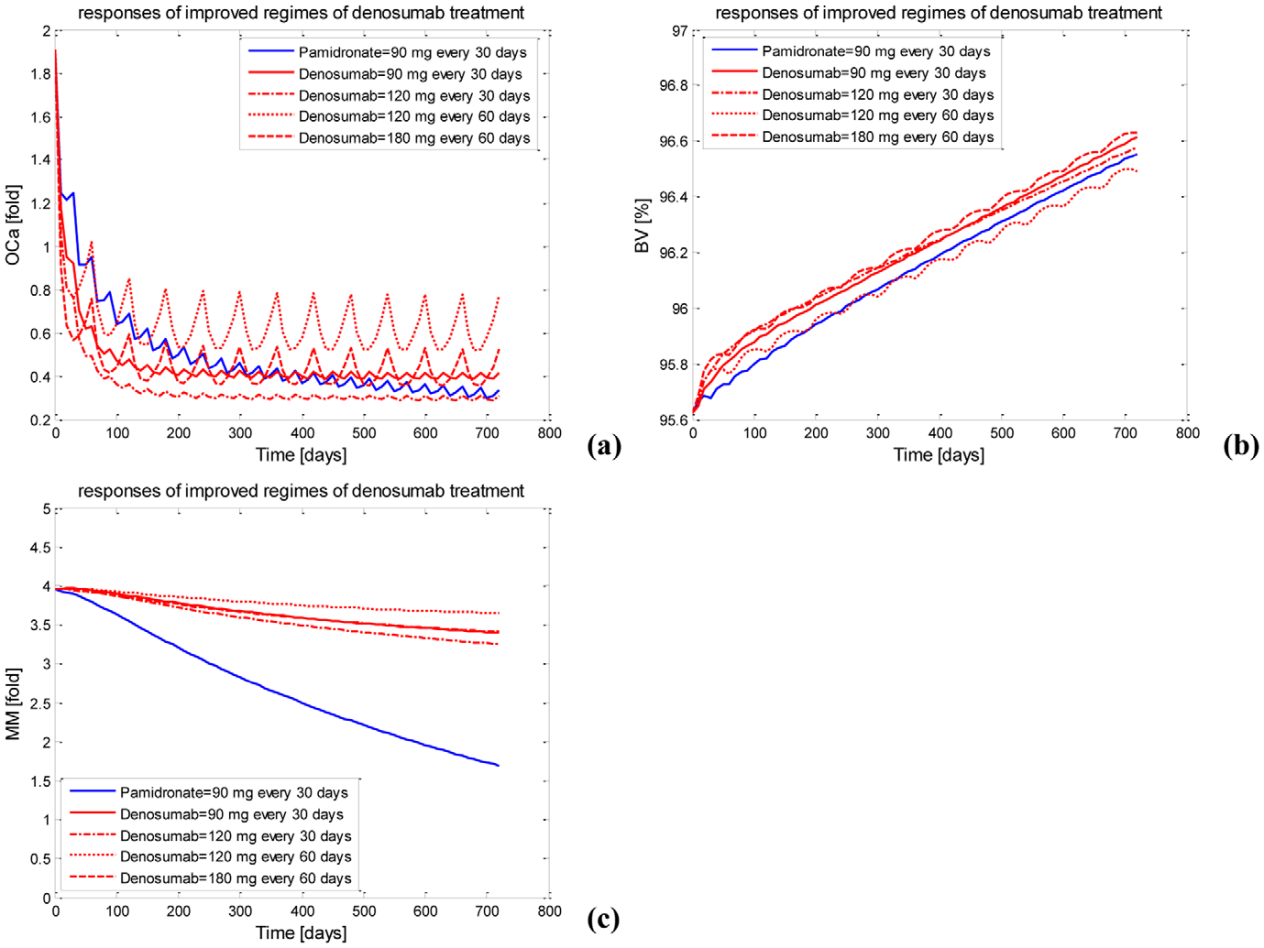
The responses using the identified denosumab regimes. (a) The active osteoclast using the identified denosumab regimes. (b) The bone volume using the identified denosumab regimes. (c) The MM-cell density using the identified denosumab regimes.

Since the different pharmacokinetics of pamidronate and denosumab mainly contribute to the different quantitative bone-related response to pamidronate and denosumab treatments, how to administrate pamidronate and denosumab (or the regime of pamidronate and denosumab) plays a key role in improving the bone-related response to pamidronate and denosumab treatments. To clarify whether denosumab represents an alternative to pamidronate in the treatment of MM-induced bone disease, the issue of whether a specific regime of denosumab produces a greater improvement in bone lesions than pamidronate has to be addressed. If a specific regime of denosumab can be found to produce a greater improvement in bone lesions than pamidronate, then denosumab may represent a viable alternative to pamidronate in the treatment of MM-induced bone disease. As a result, the above-described responses to 12 different regimes of denosumab treatments are further evaluated by the defined relative response and the relative index, resulting in the identification of four denosumab regimes that potentially produce an improved bone-related response compared with the recommended pamidronate regime. The responses of the density of osteoclasts, the bone volume and the MM-cell density to the identified denosumab regimes are shown in Figure 9.

As summarized in Table 6, the identified denosumab regimes are as follows: (i) the administration of 90 mg denosumab every 30 days; (ii) the administration of 120 mg denosumab every 30 days; (iii) the administration of 120 mg denosumab every 60 days; (iv) the administration of 180 mg denosumab every 60 days. Clearly, the relative index *Index_rela_* of these identified denosumab regimes is greater than its threshold value of 1.1. Specifically, whereas the relative response *BV_AUC,rela_* of the denosumab regimes except for the third are greater than 1, the relative response *BV_AUC,rela_* of the third denosumab regime is lower than 1. In contrast, the relative response *OC_a,AUC,rela_* of the denosumab regimes except for the third are lower than 1 but the relative response *OC_a,AUC,rela_* of the third denosumab regime is greater than 1. These differences in the relative response indicate that the denosumab regimes except for the third improve the responses of the bone volume but worsen the responses of the density of osteoclasts, whereas the third denosumab regime improves the response of the density of osteoclasts but diminishes the response of the bone volume. In other words, compared with the recommended pamidronate regime, all the identified denosumab regimes produce an overall improved bone-related response but cannot improve the responses of the bone volume and the density of osteoclasts simultaneously. In contrast, the relative response *MM_AUC,rela_* of the identified denosumab regimes is much lower than 1, as a result, the identified denosumab regimes cannot improve the MM tumor-related response in comparison with the recommended pamidronate regime because denosumab has no anti-MM action in the MM-bone compartment.

**Table 6:** The relative response and the relative index of the identified regimes of denosumab treatment.

Denosumab regime	BV_AUC,rela_	OCa_AUC,rela_	Index_rela_	MM_AUC,rela_
90 mg every 30 days	1.1157	0.9243	1.2082	0.2413
120 mg every 30 days	1.1279	0.7127	1.1991	0.3101
120 mg every 60 days	0.9823	1.3176	1.114	0.1334
180 mg every 60 days	1.1749	0.9245	1.2673	0.2459

Note 1: the pamidronate regime consisting of the intravenous administration of 90 mg pamidronate every 4 weeks for a period of 2 years is used as a base.

Clinically, the first three identified denosumab regimes are feasible because their doses are lower than or equal to the dose of the reference denosumab regime (120 mg) and their administration periods are longer than or equal to the administration period of the reference denosumab regime (every 4 weeks), suggesting that the possible toxicities (at least in terms of the risk of ONJ) caused by these denosumab regimes should be tolerable. On the other hand, the dose of the fourth identified denosumab regime (180 mg, every 60 days) is larger than that of the reference denosumab regime but the administration period of the fourth identified denosumab regime is longer than that of the reference denosumab regime; as a result, it is difficult to determine whether the fourth identified denosumab regime is feasible in the clinical setting based on the regime on its own right. However, the ratios between *BV_AUC,rela_*, *OC_a,AUC,rela_* and *Index_rela_* when the fourth identified denosumab regime is used and those when the reference denosumab regime is used are 1.0417, 1.2972 and 1.0569 respectively, indicating that the fourth identified denosumab regime is superior to the reference denosumab regime in terms of the improvements of both the bone volume and the density of osteoclasts. Therefore, the fourth identified denosumab regime is also clinically feasible. In a word, the four identified denosumab regimes not only produce an overall improved bone-related response compared with the recommended pamidronate regime but also are clinically feasible.

It should be noted that although the administration of 180 mg denosumab every 30 days produce an overall improved bone-related response (the values of *BV_AUC,rela_*, *OC_a,AUC,rela_*, *Index_rela_* and *MM_AUC,rela_* are 1.06, 0.4941, 1.1094 and 0.3876 respectively) compared with the recommended pamidronate regime, this denosumab regime is not regarded as clinical feasibility because its dose is larger than the dose of the reference denosumab regime and it induces a higher risk of ONJ than the reference denosumab regime. In addition, the above-described responses to 11 different regimes of pamidronate treatments (except the recommended pamidronate regime) are further evaluated by the relative response and the relative index. None of the evaluated pamidronate regimes can further improve the bone lesions, alleviate MM-tumor burden and decrease the risk of ONJ simultaneously compared with the recommended pamidronate regime.

## Discussion

Pharmacodynamic (PD) simulation is the current ‘gold standard’ employed by pharmaceutical companies to investigate the responses to a particular pharmacological drug; however, this approach treats the human body as a ‘black box’ or a ‘gray box’ [43] and provides no information on several important ‘local clinical variables’. In recent years, efforts have been made to model how a pharmacological drug administered to the bone microenvironment (via the circulation) can interact with the bone cells. Marathe et al. [44] recently exploited this idea by incorporating a denosumab pharmacokinetic model into a cellular bone homeostasis model to characterize the temporal profiles of the serum NTX after a one-off administration of denosumab in patients with MM. Although the simulated NTX dynamics appear to fit the experimental results quite well, an obvious limitation of this study is that the evaluation of the responses to denosumab treatment in patients with MM is based on the normal coupling between the osteoblasts and osteoclasts (in this case, bone formation and bone resorption are balanced, and no bone loss occurs). However, the activity of the osteoblasts and osteoclasts is uncoupled in patients with MM (in this case, bone formation and bone resorption are unbalanced, resulting in increased bone turnover and bone loss). This difference may undermine the reliability and accuracy of the evaluation of the responses to denosumab treatment in MM-induced bone disease. Additionally, the study of Marathe et al. [44] did not provide information with respect to the bone volume and MM-tumor burden, resulting in an incomplete evaluation. In contrast, the present study of *in silico* investigations of denosumab treatment in MM-induced bone disease is based on the previously proposed MM-bone model [29], in which the uncoupled coordination between the osteoblasts and osteoclasts has been simulated and information with respect to the bone volume and MM-tumor burden is provided. Therefore, the reliability, accuracy and completeness of the evaluation of denosumab (and pamidronate) treatments in MM-induced bone disease have the potential to be improved in this study.

The reliability and accuracy of the evaluation of pamidronate and denosumab treatments in MM-induced bone disease also depend on the calibration and validation of the integrated computational models. After multi-dose pamidronate and denosumab administration, the simulations are consistent with clinical datasets. However, after single-dose denosumab administration, the consistency between the simulations and the clinical datasets seems better qualitative (i.e., the simulation results exhibit the same trends as the clinical data) than quantitative. This effect is mainly caused by the limited clinical data available and the variability of the currently available clinical data. Using more reliable clinical data, this consistency between the simulations and the clinical data may be improved. This consistency may also be improved by refining the integrated models in the future. For example, the proposed models are deterministic whereas biological phenomena are stochastic; therefore, considering stochastic properties, the ability to capture the effects of noise may improve the differences between the best-fit modeling results and the experimental data. In addition, after single-dose pamidronate administration, the simulations only capture the sustainable decrease in NTX levels; therefore, this result may be a shortcoming of the integrated computational model that may undermine the accuracy of the evaluation of pamidronate treatment in MM-induced bone disease.

Both pamidronate and denosumab increase the bone volume by reducing the density of osteoclasts. On the other hand, the lower density of osteoclasts results in a less bone remodeling, which causes some severe side effects such as ONJ. Therefore, it is important to maintain a reasonable or balanced density of osteoclasts in pamidronate and denosumab treatments in order to increase the bone volume while decreasing the occurrence of side effects. The task to identify such a reasonable density of osteoclasts is difficult in the clinical investigations; however, it becomes easy in the *in silico* investigations. For example, the validated integrated models can be used to simulate the responses to various regimes of pamidronate and denosumab treatments. Furthermore, the defined relative indices make it possible to effectively identify the improved regime from various regimes because they concurrently contains the items of the bone volume (needs less osteoclasts) and the density of osteoclasts (need more osteoclasts). Indeed, using the integrated models and the defined relative indices, four denosumab regimes have been successfully identified, which increase the bone volume and maintain a high density of osteoclasts compared with the recommended pamidronate regime. Although it is still hard to determine whether these identified regimes are or contain the optimal regime, they are approaching to the optimal regime.

In this study, we identified four denosumab regimes that potentially produce an overall improved bone-related response compared with the recommended pamidronate regime, supporting the idea that denosumab can represent an alternative to pamidronate in the treatment of MM-induced bone lesions. Although further clinical investigations are required to verify these identified denosumab regimes, this identification greatly narrows the scope of testing regimes for clinical investigations. In this way, *in silico* investigations are complementary to clinical investigations. Clinical investigations provide the necessary clinical data for *in silico* investigations and verify the outcomes of *in silico* investigations; *in silico* investigations accelerate the process of evaluating the responses to the drug therapy and the process of screening the drug therapy regimes performed by clinical investigations. This approach of combining clinical investigations with *in silico* investigations could promote efficiency in the drug evaluation and reduce costs compared with using clinical investigations alone.

This study has demonstrated that the computational method is an efficient way to address the issue of that denosumab appears to represent an alternative agent to pamidronate in the treatment of MM-bone disease. In the meanwhile, another issue of whether denosumab can further represent an alternative agent to other bisphosphonates in the treatment of MM-bone disease arises. For example, zoledronic acid is also a widely used bisphosphonates in the treatment of MM-induced bone disease and comparing the efficacy and adverse effects of zoledronic acid with denosumab or other bisphosphonates (i.e., pamidronate) is a critical issue in the treatment of MM-bone disease. Several clinical investigations were conducted to compare the responses to zoledronic acid treatments with those to denosumab or pamidronate treatments. A randomized, double-blind study showed that denosumab (120 mg) was non-inferior (tending to superiority) to zoledronic acid (4 mg) in preventing or delaying the skeletal-related events in patients with advanced cancer metastatic to bone or MM [45]. In a phase III, double-blind comparative study, zoledronic acid (4 mg) was demonstrated to be as effective and well tolerated as 90 mg of pamidronate in the treatment of osteolytic bone lesions in patients with advanced breast cancer or MM [9]. A 25-month clinical trial confirmed the long-term safety and efficacy of zoledronic acid (4 mg) and demonstrated that zoledronic acid (4 mg) was more effective than pamidronate (90 mg) in reducing the risk of developing skeletal complications in the patients with breast cancer or MM [11]. In contrast, there were data showing that a 9.5-fold greater risk for the development of ONJ with zoledronic acid compared with pamidronate in the treatment of MM-induced bone disease, resulting in that patients with MM may prefer pamidronate to zoledronic acid [16]. In spite of advancing in clinical comparisons of zoledronic acid with denosumab and pamidronate, more comprehensive comparisons (including *in silico* comparisons) are necessary to address the issues of whether denosumab can represent an alternative agent to zoledronic acid or which one of pamidronate and zoledronic acid is superior in the treatment of MM-induced bone disease. Since this study has demonstrated that the method of *in silico* investigations is efficient, it actually paves the way for addressing the above issues by *in silico* investigations in the future study, although there may be a potential limit of this study due to not currently address the above issues in this study.

## Conclusions

In this paper, we illustrated how to investigate the anti-catabolic effects of pamidronate and denosumab treatments in MM-induced bone disease using integrated computational models that link a bone resorption marker with drug pharmacokinetics and drug actions on the MM-bone compartment. This investigation assisted in clarifying whether denosumab represents an alternative to pamidronate in the treatment of MM-induced bone lesions. To develop the integrated computational models, pharmacokinetic models of pamidronate and denosumab were developed to simulate the transient concentrations of pamidronate and denosumab respectively, and were calibrated and validated by different clinical datasets. The integrated computational models were subsequently developed by incorporating the simulated transient concentrations of pamidronate and denosumab and simulations of their actions on the MM-bone compartment into the MM-bone model. These integrated models were also calibrated and validated by different clinical datasets, suggesting that they are suitable to be applied to investigate the responses to pamidronate and denosumab treatments in MM-induced bone disease.

Using validated integrated models, a variety of regimes of pamidronate and denosumab treatments were investigated, and the corresponding responses were evaluated by the defined relative responses and the relative index, which are based on the quantification of the responses to pamidronate and denosumab treatments. The outcomes of the evaluation identified four denosumab regimes that potentially produce an overall improved bone-related response compared with the recommended pamidronate regime. This identification supports the idea that denosumab can represent an alternative to pamidronate in the treatment of MM-induced bone disease.

This study indicates that *in silico* investigations are valuable as a complementary approach to clinical investigations. This approach substantially accelerated the process of evaluating the responses to drug therapy and the process of screening drug therapy regimes, leading to the identification of several improved regimes with reduced costs in terms of time and money. In combination with clinical investigations, the proposal of improved regimes may be specifically tested and verified. As a result, clinical investigations combined with *in silico* investigations have the potential to transform the traditional method of pure screening by clinical investigations, leading to a new approach of mixed screening by *in silico* investigations and verification by clinical investigations. This combined approach promotes the efficiency of drug evaluation and reduces the costs. In addition, based on the MM-bone model, the method of *in silico* investigations of the anti-catabolic effects of pamidronate and denosumab on MM-induced bone disease may be utilized to investigate the anti-catabolic effects of other bisphosphonates (i.e., zoledronic acid) or the anabolic effects of novel agents (i.e., bortezomib) on MM-induced bone disease.

## Supporting Information

Supporting Information S1 The derivation of *K_D,Den_*.
(DOC)

Supporting Information S2 The calculations of the area under the curves (AUCs) for the bone volume, the MM-cell density and the density of active osteoclasts.
(DOC)

Supporting Information S3 The smoothed curves of chemotherapy and the combined therapy (chemotherapy + pamidronate).
(DOC)
