# Supplementary material for: In Silico Investigations of the Anti-Catabolic Effects of Pamidronate and Denosumab on Multiple Myeloma-Induced Bone Disease
Source: PLoS One. 2012 Sep 21;7(9):e44868. doi: 10.1371/journal.pone.0044868 (PMC3448612; doi:10.1371/journal.pone.0044868)
Supplement: Supporting Information S1 — The derivation of KD,Den. (DOC) [file pone.0044868.s001.doc]

With denosumab treatments, the binding of denosumab to RANKL influences the concentrations of RANKL. By assuming that denosumab proportionally diffuses from the serum to the bone, the concentrations of RANKL are calculated by the following:

$$RANKL=\frac{P_{RANKL,d}+\beta_{RANKL}\cdot OB_{p}}{(1+K_{A,OPG}\cdot OPG+K_{A,RANK}\cdot RANK+\frac{m_{Den,2}\cdot\tilde{f}_{Den,2}}{\tilde{K}_{D,Den}})\cdot(\frac{\beta_{RANKL}}{R^{RANKL}\cdot\pi_{RANKL}^{ligands}}+D_{RANKL})}$$

where, *m_Den,2_* is the denosumab concentration in serum and $\tilde{f}_{Den,2}$ is a percentage factor to represent the diffusion of denosumab from the serum to the bone. $\tilde{K}_{D,Den}$ is the dissociation rate constant of denosumab binding to RANKL. Without changing the effects of denosumab on the bone resorption, the calculation of RANKL concentrations is simplified by setting $K_{D,Den}=\tilde{K}_{D,Den}/\tilde{f}_{Den,2}$. As a result, the Eq.(S1) is reduced to the Eq.(6) and *K_D,Den_* is actually a composite parameter.
