# Supplementary material for: In Silico Investigations of the Anti-Catabolic Effects of Pamidronate and Denosumab on Multiple Myeloma-Induced Bone Disease
Source: PLoS One. 2012 Sep 21;7(9):e44868. doi: 10.1371/journal.pone.0044868 (PMC3448612; doi:10.1371/journal.pone.0044868)
Supplement: Supporting Information S2 — The calculations of the area under the curves (AUCs) for the bone volume, the MM-cell density and the density of active osteoclasts. (DOC) [file pone.0044868.s002.doc]

The specific calculations of the area under curves (AUCs) for the bone volume, the MM-cell density and the density of osteoclasts (denoted by *BV_AUC_*, *MM_AUC_* and *OCa,_AUC_* respectively) are illustrated in Figure S1.

Figure S1. The calculations of the area under the curves (AUCs) for the bone volume, the MM-cell density and the density of active osteoclasts.
