# Supplementary material for: In Silico Investigations of the Anti-Catabolic Effects of Pamidronate and Denosumab on Multiple Myeloma-Induced Bone Disease
Source: PLoS One. 2012 Sep 21;7(9):e44868. doi: 10.1371/journal.pone.0044868 (PMC3448612; doi:10.1371/journal.pone.0044868)
Supplement: Supporting Information S3 — The smoothed curves of chemotherapy and the combined therapy (chemotherapy + pamidronate). (DOC) [file pone.0044868.s003.doc]

To smooth the two curves (paraproteinchanges_chemo+pami_ and paraproteinchanges_chemo_), the curve fittings are conducted by using the interactive curve fitting tool “cftool” in Matlab. The exponential models are used in the curve fittings and the goodness of the fitting is evaluated by r^2^. The smoothed curves are displayed in Figure S1 and r^2^ of the red and blue curves are 0.6026 and 0.9661 respectively. Furthermore, the Eq.(S2) and (S3) show the specific fitting models of the red and blue curves respectively.

Figure S1. The smoothed curves of chemotherapy and the combined therapy (chemotherapy + pamidronate).

$$y=100.6\times e^{-0.0008946\times x}$$

$$y=97.59\times e^{-0.00249\times x}$$
